# A Mechanistic Prediction Model of Resistance to Uprooting of Coniferous Trees in Heilongjiang Province, China

**DOI:** 10.3390/plants13172377

**Published:** 2024-08-26

**Authors:** Guangqiang Xie, Yaoxiang Li, Lihai Wang, Xiangcheng Kan, Ping Zhang

**Affiliations:** 1 College of Mechanical and Electrical Engineering, Northeast Forestry University, Harbin 150040, China; xieguangqiang@nefu.edu.cn (G.X.); kxc@nefu.edu.cn (X.K.); ping.zhang@wur.nl (P.Z.); 2 School of Automotive and Electromechanical Engineering, Harbin Cambridge University, Harbin 150069, China

**Keywords:** wind damage, root plate, GPR, resistance to uprooting, mechanistic model

## Abstract

Coarse roots and the root plate play an important role in tree resistance to uprooting. In this study, a qualitative mechanistic model was developed to analyze coniferous tree resistance to uprooting in relation to tree morphological characteristics. The sizes of the crown, stem, and root plate of twenty sample spruces and twenty sample Korean pines were individually measured for this purpose. Using Ground Penetrating Radar (GPR), the coarse root distribution and root plate size were detected. In the qualitative mechanistic model, a larger crown area increased the overturning moment, while higher DBH and root plate mass increased the resistance moment. The resistance coefficient (*R_m_*) was calculated by comparing resistive and overturning moments, classifying samples into three uprooting hazard levels. Trees with smaller crown areas, larger stems, and root plates tend to have higher resistance to uprooting, as indicated by higher *R_m_* values. This qualitative mechanistic model provides a useful tool for assessing coniferous standing tree uprooting resistance.

## 1. Introduction

Forests constitute the largest terrestrial ecosystem and play a crucial role in maintaining eco-safety while fostering sustainable development of the economy and society. The Heilongjiang Province Forest region, one of China’s primary forested areas, has abundant forest resources. However, abiotic hazards currently pose the most significant risk in forest management, with wind recognized as the principal abiotic factor causing forest damage [1]. Coniferous forests, as the main forest stands in Heilongjiang Province, are more vulnerable to wind damage than broadleaved forests [2]. Specifically, Korean pine and spruce, which are the significant coniferous species in the natural forests of Heilongjiang Province, are more susceptible to uprooting compared to others [3,4]. Strong winds can lead to tree deflection exceeding critical stability thresholds, resulting in substantial tree mortality in Heilongjiang Province due to wind-forest interactions [3]. Wind damage not only poses significant challenges to forest operations but also affects strategic aspects, such as logistics, labor, and storage capacity, as well as economic aspects such as reducing timber prices and additional costs for replanting [5].

Uprooting, identified as the most significant form of wind-induced forest damage [6], entails the process of a tree falling where a portion or the entirety of the root system is broken off from the soil by external forces [7,8,9]. Additionally, the uprooted trees may spread dangerous fungal bacteria or pests to the surviving trees, potentially leading to more severe and prolonged damage to standing trees [10,11]. Hence, the assessment of tree resistance to uprooting is crucial in reducing the risk of wind damage to coniferous forests.

Previous studies have highlighted tree morphological characteristics as significant factors influencing tree resistance to uprooting, including tree height, diameter at breast height (DBH), spread crown area, root distribution and biomass, and root plate size and mass [12,13,14,15]. Various physical models have been developed to predict the trend of tree resistance to uprooting based on tree aboveground shapes [16,17,18]. However, due to the challenges in detecting the distribution and biomass of coarse roots, the effect of root plate size and mass on tree resistance to uprooting has not been further analyzed.

Tree roots, essential organs of standing trees, provide stability and support for the tree’s upright structure [19]. However, detecting root distribution is challenging compared to the stem and crown due to the roots’ specific growth areas. In woody vegetation, tree roots are generally classified into coarse root and fine roots, based on the root size [20,21]. Fine roots, defined as less than 2 mm in size [22,23], function primarily in nutrient and water absorption. Coarse roots, which store most of the carbon in the root system, are mainly responsible for anchoring and supporting the aboveground parts and transporting resources to the stem [24]. Despite studies on the mechanisms that indicate that the critical role of coarse roots and the root plate is in resistance to uprooting, these have been mainly statistical [25,26,27]. Statistical approaches do not define the causal links between underground morphological characteristics and resistance to uprooting, which can be explicitly described using a mechanistic approach.

Unfortunately, the uncertainties in the soil and the complexity of the root structure make the detection of coarse roots and assessment of the root plate extremely challenging. Several methods, such as excavation sampling of whole roots, trench, and soil core, were used for the study of coarse roots [28,29,30,31,32,33]. However, these excavation methods are labor intensive, time-consuming, and costly. Additionally, they often cause substantial damage to trees and the surrounding soil environment, rendering long-term dynamic observation impractical.

Ground Penetrating Radar (GPR), a non-destructive detection method, is widely used for coarse root detection [34]. Hruska et al. [35] pioneered the application of GPR for root distribution detection, yielding satisfactory results. With the development of GPR, this technique is being applied to root detection by more and more scholars. At present, GPR has been widely used in root detection for large areas [20,36], including the spatial distribution of coarse roots [35,37,38] and the size, biomass, and volume estimations of coarse roots [39,40,41,42]. Given the soil condition uncertainties in the Heilongjiang Province forest region, there have been fewer studies of coarse root distribution in this area. Therefore, it is necessary to accurately detect the distribution of coarse roots and measure the volume of the root plate using GPR.

The purpose of this study was to assess tree resistance to uprooting. For this purpose, a qualitative mechanistic model of tree resistance to uprooting was developed based on variations in tree morphological characteristics (crown size, stem size, and root plate size and mass) and was analyzed using static analysis. The coefficient of resistance to uprooting was determined based on the ratio of total resistive moments to the overturning moment. All samples were classified into three hazard levels of uprooting, according to the coefficient. This study aims to provide forest managers with a predictive model for rapidly assessing the hazard levels of the uprooting of standing trees.

## 2. Results

### 2.1. Coarse Root Distribution

**Figure 1:**
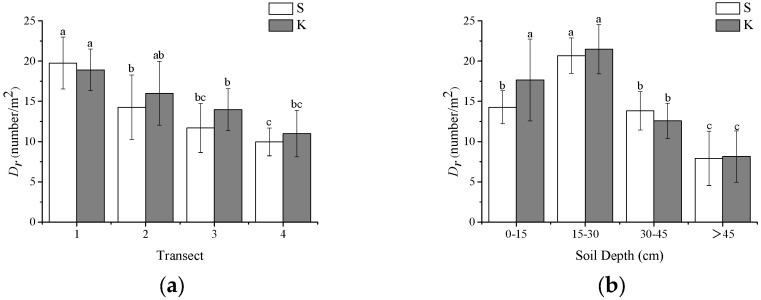
Root density distribution in different directions. (**a**) horizontal distribution in different transects; (**b**) vertical distribution with varied soil depth. S: spruce, K: Korean pine. According to Tukey HSD tests, values under different letters are significantly different with a probability of *p* < 0.05.

The distribution of coarse roots was reflected by coarse root density, which varies in the vertical and horizontal directions. As shown in Figure 1a, in the horizontal direction, the average densities of coarse roots for spruce and Korean pine decrease as the scanning radius increases. The average root densities of Korean pine and spruce are highest at transect 1, with a noticeable decline in average densities from transect 2 onwards. Based on the results of the Tukey HSD tests, the average coarse root densities are significantly different between transects 1 and 3. However, the average coarse root densities of Korean pine and spruce have an insignificant change between transects 3 and 4. This indicates that as the distance from the stem increases, the number of coarse roots decreases. The variations in coarse root density horizontally are mainly observed between transects 1 and 3.

The average coarse root densities in the vertical direction are presented in Figure 1b. Korean pine and spruce exhibit the highest root densities within the 15–30 cm depth range. Coarse root density increases with soil depth between 0 and 30 cm, but decreases beyond 30 cm. Korean pine’s average coarse root density within the 15–30 cm depth range does not significantly differ from that within the 0–15 cm range, but significantly differs from depths beyond 30 cm. Spruce’s coarse root density within the 15–30 cm depth range significantly differs from both the 0–15 cm and 30–45 cm ranges. These results indicate that the middle layer of the root system primarily reflects variations in coarse root density. Moreover, over 70% of Korean pine’s coarse roots are distributed within the 0–30 cm depth range, surpassing spruce’s density in the same range.

### 2.2. Soil Properties and Root Plate Mass

The data in Table 1 shows clear trends in soil properties across different depths. Bulk density decreases initially and then increases with depth, reaching its minimum at 15–30 cm. Porosity increases initially, then decreases and slightly increases at greater depths. Water content follows a similar pattern, increasing initially and then decreasing with depth. Porosity and water content peak at 15–30 cm. In terms of soil texture, sand content decreases initially and then slightly increases at greater depths, silt content peaks at mid-depth, and clay content consistently increases with depth.

When comparing the shallow depths (0–30 cm) with deeper depths (>30 cm), the shallow layers generally exhibit lower bulk density, higher porosity, and higher water content. In contrast, the deeper layers tend to have higher bulk density, lower porosity, and lower water content. Additionally, the sand and silt content are higher in the shallow layers, while the clay content increases with depth. Soil penetration resistance in the 0–30 cm depth range was lower than that at a depth beyond 30 cm; therefore, coarse root density within the 0–30 cm depth range was much higher than that beyond the 30 cm depth range for all samples (Figure 1b).

The volume of the root plate of all samples was determined from the detection results of TRU, and then the root plate mass of all samples was calculated according to Equations (5)–(8) (Table 2). The root plate masses of all samples ranged from 319.62 to 5650.87 kg, with the coarse root biomass accounting for 2–20% (17.53–1150 kg) and a mean value of 5.49% of the root plate mass for all samples. Additionally, the root plate volume of all samples ranged from 0.28 to 4.18 m^3^. The soil mass within the root plate ranged from 302.04 to 4556.2 kg, which is much greater than the coarse root biomass.

Root plate volume and mass varied between both tree species. The mean coarse root biomass, soil mass, and root plate volume of spruce were lower than those of Korean pine. This is similar to the difference in DBH between spruce and Korean pine. The average DBH of spruce and Korean pine is 35.1 cm and 42.9 cm, respectively. The coarse roots grow with the age of the tree, resulting in a larger root plate [43].

### 2.3. Assessment of Samples in Resistance to Uprooting Using Data on Tree Morphological Characteristics

Firstly, the $\Delta^{\prime }$ of all samples was calculated using Equation (14). The $\Delta^{\prime }$ of the uprooted tree is assumed to be the critical uprooting threshold. To determine the critical uprooting threshold for spruce and Korean pine, the morphological characteristics data of uprooted trees from Ge et al. [3] research were reanalyzed using Equation (14). The characteristics of the study sites and forest structure selected in Ge’s research are similar to those in this study. Ge’s research investigated 20 uprooted spruces and 60 uprooted Korean pines. The average morphological characteristics of these 80 uprooted trees are shown in Table 3.

The ${\Delta^{\prime }}_{u}$ for 60 uprooted spruces and 20 uprooted Korean pines was computed, yielding an average critical uprooting threshold $\bar{{\Delta^{\prime }}_{u}}$ of 0.66 ± 0.33 and 0.46 ± 0.38 for spruce and Korean pine uprooting, respectively. The 95% confidence level for ${\Delta^{\prime }}_{u}$ of spruce was 1.31, and the 99% confidence level of spruce was 1.51. Similarly, the 95% confidence level for ${\Delta^{\prime }}_{u}$ of Korean pine was 1.20, and the 99% confidence interval of Korean pine was 1.44. Therefore, this study classifies the spruce samples into three hazard levels of uprooting high ($\Delta^{\prime }$ ≤ 1.31), moderate (1.31 < $\Delta^{\prime }$ ≤ 1.51), and low (1.51 < $\Delta^{\prime }$), while the Korean pine samples were classified into three hazard levels of uprooting high ($\Delta^{\prime }$ ≤ 1.20), moderate (1.20 < $\Delta^{\prime }$ ≤ 1.44), and low (1.44 < $\Delta^{\prime }$).

Samples of spruce and Korean pine with a larger $\Delta^{\prime }$ compared to 1.51 and 1.44, respectively, are expected to be more stable and resistant to uprooting or have a lower uprooting risk level. Samples of spruce and Korean pine with a $\Delta^{\prime }$ less than 1.31 and 1.20, respectively, are initially identified as having a high hazard level of uprooting, with a high probability of uprooting in wind damage events. The $\Delta^{\prime }$ values of all samples and the resulting hazard levels of uprooting are presented in Table 4. There are 12 samples of spruce and 13 samples of Korean pine classified as having a high hazard level of uprooting. In the event of wind damage, these 25 samples are highly likely to be uprooted. There are two samples of spruce and two samples of Korean pine classified as having a moderate hazard level of uprooting. Despite not having a high hazard level of uprooting, they still have the risk of uprooting in the event of wind damage.

## 3. Discussion

Coarse roots are called skeleton roots, which provide support for the tree against strong winds and pressure [44]. From the results of the GPR detection, the horizontal distribution range of coarse roots is consistently larger than the vertical distribution range across all samples. Hence, the coarse root distributions of all samples are in accordance with the horizontal distribution characteristics [45,46].

The characteristics of the coarse root horizontal distribution are primarily influenced by soil properties. Similar to the stem, the root system expands with age, leading to an increase in both the diameter and length of coarse roots [47]. Specifically, as trees grow, their demand for nutrients and water grows in tandem with the expansion of the crown and stem. Moreover, the increasing weight and height of the aboveground parts further exacerbate this demand. However, as soil depth increases, soil strength rises, limiting the ability of the plant roots to penetrate the soil [48]. Deeper soil layers exhibit a notable decrease in soil porosity and an increase in bulk density. Penetration resistance of the soil also increased with clay content. These conditions pose challenges for coarse root extension into deeper soils, thereby promoting horizontal root growth [49]. In addition, root distribution is related to the climate [50]. The study area experiences a humid and cold climate, characterized by shallow, acidic soil layers and high levels of underground water, all of which promote shallow root distributions.

The root plate mass was influenced by both the extent of the coarse root distribution and the soil properties. As the DBH increased, coarse root elongation and root plate development continued to progress [51]. However, despite some samples having similar DBH sizes (e.g., K2 and K19), significant differences were observed in their root plate mass and volume due to the high penetration resistance of deeper soils, limiting the elongation of coarse roots. Soil properties in the sample plots varied, and thus when some samples grew in relatively hard soil, their root plate volume and mass might have been smaller than those of other samples. The proportion of coarse root biomass in the root plate could indicate root plate formation. If the soil has a high bulk density, a relatively small quantity of coarse roots can hold a high soil mass. Instead, low porosity could restrict the downward development of the root plate and taproot, thereby reducing anchorage efficiency [52].

In this study, a qualitative mechanistic model was developed based on the tree’s morphological characteristics. Using the *R_m_* and $\Delta^{\prime }$, the samples were classified into three hazard levels of uprooting. Unlike previous methods that relied solely on the slenderness ratio, which overlooks the influence of root plates [53,54,55], this model considers the root plate’s effect on tree stability. Variations in root plate size can lead to different uprooting hazard levels, even with the same slenderness ratio. Additionally, the choice of dependent variables in this model, including crown, stem, and root plate characteristics, differs from previous studies [16], which typically focused on above-ground morphological features.

The significance of each component in the model is highlighted by previous research. Specifically, the tree crown, as the major part of the wind load, plays a crucial role in the tree’s resistance to uprooting. Dunham et al. [56] indicated that increasing crown area can decrease tree stability, emphasizing the importance of crown size in assessing the uprooting risk. Furthermore, the stiffness of the stem is highly correlated to the wind stability of the tree. Fredericksen et al. [57] found a significant relationship between the windfall-resisting moment of Loblolly pine (*Pinus ponderosa*) and stem volume, diameter at breast height, and tree height, which proves that larger stems contribute to greater resistance against uprooting. Cannon et al. [58] found that the tree resistance to uprooting increased with the size of the stem, and the resistant moment of the stem increased with DBH and stem volume.

The root plate, often overlooked in previous models, emerges as a critical component in this qualitative mechanistic model. It serves as a vital organ for supporting the tree, with its mass and volume playing essential roles in resistance to uprooting [17,59]. Coarse root systems of uprooting-resistant trees are structured into a rigid “cage” comprising coarse roots, fine roots, and sediments that firmly hold a significant amount of soil [60]. This cage rarely breaks during uprooting. For standing trees with a horizontal coarse root distribution, soil mass is more important than soil strength [61]. The soil mass within the root plate far exceeds the coarse root biomass, with soil being the primary constituent of the root plate. When the volume of the root plate is insufficient to contain an ample amount of heavy soil, the root plates fail to provide adequate anchorage for trees, thereby reducing their stability against uprooting [62].

The model’s classification into three hazard levels based on morphological characteristics allows for a rapid initial assessment of uprooting risk, providing an efficient method for forest managers to evaluate standing tree resistance to uprooting. Forest managers can use this method to develop different responses to different hazard levels of standing trees. For example, trees categorized with a high hazard level of uprooting may be promptly harvested to prevent damage to other standing trees post-uprooting. The crown of trees classified with a moderate hazard level of uprooting could be pruned to reduce the hazard level. Additionally, this model provides a novel research framework for evaluating tree resistance to uprooting in urban environments, which may potentially enhance research efforts to improve public safety and property protection.

## 4. Materials and Methods

### 4.1. Study Site

The study is conducted at Xinghuo Forest Farm, Harbin City, Heilongjiang Province, China (45°73′ N, 129°24′ E). Located in the warm temperate zone with a semi-humid monsoon climate, it is characterized by low and gentle topography ranging from 300 m to 500 m. The annual average temperature is 10.5 °C with a mean annual precipitation of 579.7 mm. The study site supports a coniferous-broadleaved mixed plantation of the temperate zone characterized by spruce (*Picea asperata*) and Korean pine (*Pinus koraiensis*). Other tree species are white birch (*Betula platyphylla*), aspen (*Populus davidiana*), maple (*Acer mono*), and Manchurian ash (*Fraxinus mandshurica*). The soil is typically dark brown forest soil with a loam texture and the major clay mineral of hydromica. The soil profile is an O horizon of 2–6 cm, A horizon of 6–20 cm, B horizon of 20–73 cm, and C horizon of 73–106 cm. The relatively fine-textured clay soil in the study site has high organic matter content, high water-holding capacity, and a fine drainage effect [63]. Before the experiment, a preliminary trial of root detection using GPR was conducted to ensure the feasibility of obtaining bright clear images of coarse roots.

### 4.2. GPR Equipment and Data Acquisition in the Field

**Figure 2:**
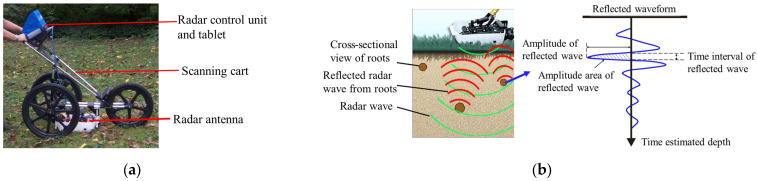
Overview of the TRU. (**a**) Components of TRU. (**b**) A schematic diagram of TRU.

The GPR used in this study was the Tree Radar Unit (TRU), which was obtained from TreeRadar Inc. (Silver Spring, MD, USA). It consisted of a field data manager and a 900 MHz radar antenna, providing a precision of 1 cm diameter within a soil depth of 1 m (Figure 2). This frequency enables accurate detection of the coarse roots of standing trees while minimizing interference from ground vegetation and shrubs.

**Figure 3:**
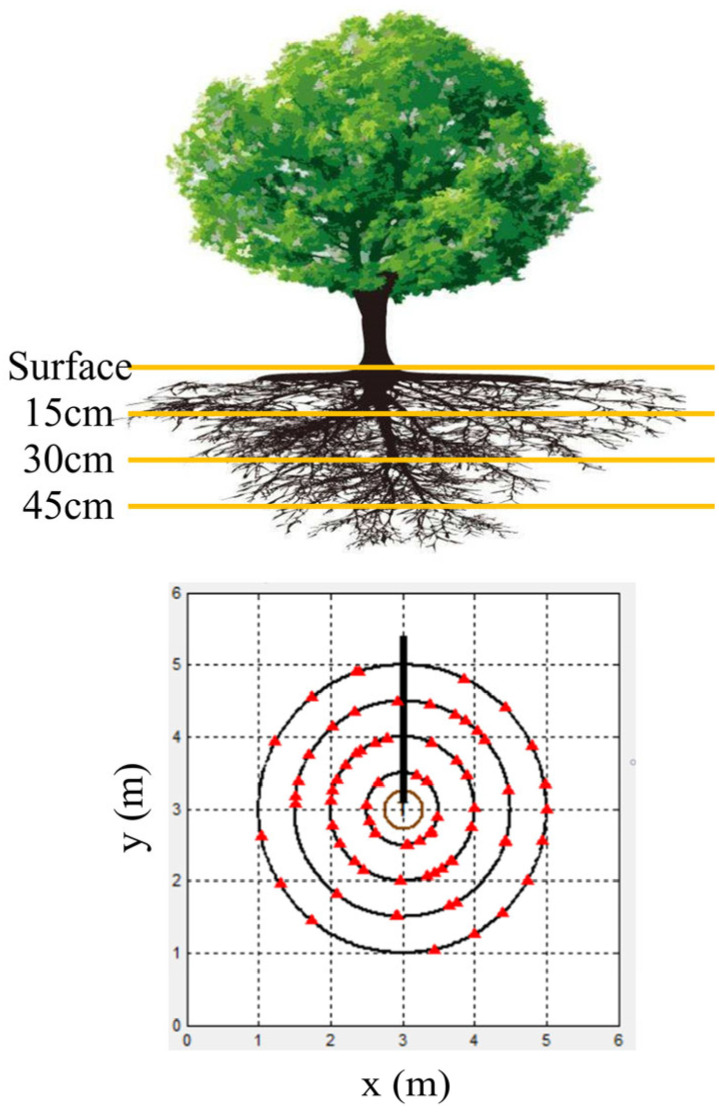
Experimental design of coarse root detection using TRU.

To assess the resistance of standing trees to uprooting, a 2.5-hectare plot was delineated in a coniferous-broadleaved mixed plantation. This plantation has a standing density of 776 plants per hectare and a canopy density of 0.65%. This plot has a southeast aspect and a hillslope gradient of 6°. The dominant species in the plot are spruce and Korean pine. Twenty standing sample spruces (labeled S1 to S20) and twenty standing sample Korean pines (labeled K1 to K20) were selected within the plot. The specific above-ground morphological characteristics of the sample trees are shown in Table 5. The coarse roots of each sample were detected at horizontal radii of 0.5 m, 1.0 m, 1.5 m, and 2.0 m (transect 1 to 4), as well as at vertical depth ranges of 0–15 cm, 15–30 cm, 30–45 cm, and deeper than 45 cm (Figure 3).

### 4.3. Soil Physical Properties

To measure the physical properties of the soil (volumetric water content, bulk density, porosity), soil samples were collected from four layers: 0–15 cm, 15–30 cm, 30–45 cm, and deeper than 45 cm. Samples were obtained at three points along four transects using a cutting ring for physical analysis [64]. Specifically, soil gravimetric moisture content was measured by oven-drying soil samples at 105 °C for 24 h. Soil bulk density was estimated from the weight and volume of soil cores after deducting those of rocks and plant roots, and then soil porosity was calculated with an assumed soil particle density of 2.65 g cm^−3^. Soil texture was classified as defined by the USDA method [65].

### 4.4. GPR Data Collection and Analusis

**Figure 4:**
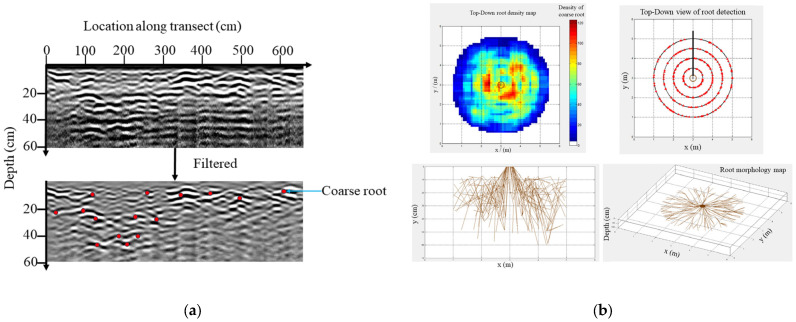
Postcollection data processing. (**a**) processing of filtered and Hilbert transformation. (**b**) data analysis.

The B-scan images of TRU were collected and processed using TreeWin software. All B-scan images underwent post-processing steps, including position correction and background removal. Additionally, radar profile normalization and filtration routines were applied to eliminate soil horizon and surface horizontal reflections. The position of coarse roots was identified manually, based on automatic software identification [66] (Figure 4). According to their positions, the 3D distribution of coarse roots was mapped with TreeWin 104b-K and TBA 2013b software.

Additionally, the coarse root density was defined as the number of coarse roots per unit area, calculated using the known scanning length and the short axis of the radar-formed ellipse [67]. Analysis of variance (ANOVA) was used to analyze the differences in average coarse root density at different transects and different depths, and a Tukey–HSD test was used to analyze further differences between classes. SPSS (version 19.0; SPSS, 2010) was used for all statistical analyses.

### 4.5. Mechanistic Model of Tree Resistance to Wind-Included Uprooting

**Figure 5:**
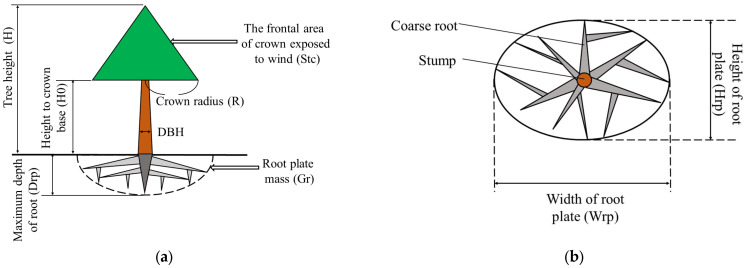
Model tree structure. (**a**) Vertical view showing the crown, stem, and root plate. (**b**) Top view of the root plate.

In this study, a mechanistic model of tree resistance to uprooting in relation to tree morphological characteristics was developed. The model tree was divided into three main parts: crown, stem, and root plate. The tree morphological characteristics were closely related to the hazard level of uprooting. The root plate, stem size, and crown size were used to describe the tree’s morphological characteristics (Figure 5). The assumptions made in the model are as follows:

1. The tree is upright and healthy, with the root plate strong enough to support the aboveground components in windless conditions. The shape of the crown is conical. The anchorage point of the tree under wind load conditions is at the base of the stem.
2. The wind load is applied horizontally and only directly to the tree crown with the assumption that the wind speed (*v*) is constant. The wind load is positively proportional to the frontal area of the crown (*S_tc_*) [16]. The center of gravity of the wind load is located at 2/3 of the crown length from the crown top [68,69]. The overturning moment of the wind acts on the anchorage point of the tree through the stem.
3. Due to elasticity, the stem bends when wind loads are applied to the crown of the tree. However, some of the wind load can be offset by the elastic restoring force of the stem, which is positively correlated with the stiffness of the stem. The most significant factors affecting the resistive moment of the stem are tree height and DBH [55,70,71,72].
4. Tree resistance to uprooting is influenced by the support provided by the root plate anchorage, which depends on a combination of four factors: the root plate mass, the strength of the windward roots and root hinge, and the soil strength at the base of the plate [26]. The mechanistic model calculates the resistive moment of the root plate based on the predicted root plate mass, as the contributions of factors other than root plate mass are very complex. The percentage contribution of root plate mass to root anchorage is constant [73,74]. Therefore, the resistive moment of the root plate can be calculated from the root plate mass, as demonstrated in the study by Peltola et al. [55]. Considering the complexity of the tree’s underground component, the root plate, which consists of coarse roots and surrounding soil, is conceptualized as a half-ellipsoid. The center of gravity of the root plate is located at 4 *D_rp_*/3π of the depth from the bottom of the root plate [75]. The mass of the root plate is mainly composed of the mass of the coarse root (*G_cr_*) and the mass of the soil in the root plate (*G_s_*). *G_s_* is proportionally correlated to the bulk density of soil ($\rho_{s}$) and the volume of the root plate (*V_r_*) [62,76].

Based on the provided assumptions, *S_tc_* can be obtained as follows:

(1) $$S_{tc}=R(H-H_{0}),$$

where *R* is the crown radius, *H* is the tree height, and *H*_0_ is the height to the crown base.

The wind load on the crown (*W_c_*) is determined as:

(2) $$W_{c}=0.5\rho_{a}C_{d}R(H-H_{0})v^{2},$$

where $\rho_{a}$ is the density of the air (a constant > 0). *C_d_* is the drag coefficient [68,69].

Therefore, the moments acting on the anchorage points of the tree are the overturning moment (*M_w_*), which was determined as:

(3) $$M_{w}=W_{c}L=0.5\rho_{a}C_{d}R\left(H-H_{0}\right)v^{2}\cdot \frac{H+2H_{0}}{3}=\frac{\rho_{a}C_{d}Rv^{2}}{6}\left(H+{2H}_{0}\right)\left(H-H_{0}\right),$$

Based on assumption 3, the resistive moment of the stem (*M_s_*) was related to DBH, as shown in Peltola et al. [55].

(4) $$M_{s}=aD^{3}H,$$

where *H* is tree height and *D* is DBH. The a values for Korean pine and spruce are 3.589 and 3.315, respectively. Furthermore, it was assumed that the wind would not be able to break the stems.

Based on assumption 4, the half-ellipsoid model of Denny et al. [77] and Norman et al. [78] was used to calculate the *V_r_*,

(5) $$V_{r}=\frac{2}{3}\pi \left(\frac{D_{rp}H_{rp}W_{rp}}{2}\right),$$

The *G_s_* was determined as:

(6) $$G_{s}=\rho_{s}V_{r},$$

Developing an accurate predictive model for coarse root biomass using GPR is challenging due to the complexity of the soil. Therefore, in this study, different nonlinear models [4] were used to calculate the coarse root biomass of spruce and Korean pine, respectively.

The nonlinear equations for coarse root biomass of spruce and Korean pine are as follows:

(7) $$G_{Scr}=4.3020D^{2.9132}H^{-0.3292},$$

(8) $$G_{Kcr}=4.7202D^{2.7794}H^{-0.0536},$$

where $G_{Scr}$ and $G_{Kcr}$ are the coarse root biomass of spruce and Korean pine, respectively. 

Based on assumption 4, the resistive moment of root plate (*M_r_*) was related to root plate mass, as shown in Peltola et al. [55].

(9) $$M_{r}={f_{r}\cdot L_{r}\cdot G}_{r}\cdot g=\frac{4{f_{r}D}_{rp}}{3\pi }\left(G_{cr}+G_{s}\right)\cdot g=\frac{4{f_{r}D}_{rp}}{3\pi }\left(G_{cr}+\rho_{s}V_{r}\right)\cdot g,$$

where *g* is the gravitational constant (9.81 N/kg). $f_{r}$ is a dimensionless parameter (a constant > 0) representing root plate weight as a percentage of total root anchorage. The soil type in the plot is dark brown loam, and $f_{r}$ of spruce and Korean pine are 20% and 30%, respectively, based on the results from Peltola et al. [74].

Let the coefficient of resistance to uprooting (*R_m_*) be the ratio of the total resistive moment to overturning moment, and *R_m_* is determined as:

(10) $$R_{m}=\frac{M_{s}+M_{r}}{M_{w}},$$

Thus, the tree becomes more resistant with increasing *R_m_*, and vice versa.

Based on assumptions 1 and 2, assuming that:

(11) $$\mu =\frac{\rho_{a}C_{d}v^{2}}{6},$$

where μ is a constant (>0).

Substituting μ into Equation (3) results in:

(12) $$M_{w}=W_{c}L=\mu R\left(H+{2H}_{0}\right)\left(H-H_{0}\right),$$

Therefore,

(13) $$R_{m}=\frac{M_{s}+M_{r}}{M_{w}}=\frac{1}{\mu }\left(\frac{aD^{3}H\cdot }{R\left(H+2H_{0}\right)\left(H-H_{0}\right)}+\frac{4{f_{r}D}_{rp}\left(G_{cr}+\rho_{s}V_{r}\right)\cdot g}{3\pi R\left(H+{2H}_{0}\right)\left(H-H_{0}\right)}\right)\;=\frac{1}{\mu }\cdot \frac{3\pi aD^{3}H+4f_{r}D_{rp}\left(G_{cr}+\rho_{s}V_{r}\right)\cdot g}{3\pi R\left(H+2H_{0}\right)\left(H-H_{0}\right)},$$

According to Equation (13), assuming that:

(14) $$\Delta^{\prime }=\frac{3\pi {aD}^{3}H+4f_{r}D_{rp}\left(G_{cr}+\rho_{s}V_{r}\right)\cdot g}{3\pi R\left(H+{2H}_{0}\right)\left(H-H_{0}\right)},$$

The *R_m_* increases with the increase of $\Delta^{\prime }$ because μ is a constant (>0). Therefore, the assessment of resistance to uprooting of trees could be well characterized with $\Delta^{\prime }$.

## 5. Conclusions

This study presents a novel qualitative mechanistic model for evaluating tree resistance to uprooting based on morphological characteristics. Firstly, coarse root distribution was estimated using GPR. The root plate volume was calculated based on the edges of the coarse root distribution. The mass of the root plate was obtained by calculating the coarse root biomass and the mass of the soil within the root plate. The results indicate that the coarse root distributions of all samples are in accordance with the horizontal distribution characteristics. Soil mass is the primary component of root plate weight, offering significant anchorage for trees. A qualitative model for tree resistance to uprooting was established using the overturning moment and resistive moment, with *R_m_* and $\Delta^{\prime }$ calculated based on their respective ratios. Based on $\Delta^{\prime }$, all samples were classified into three hazard levels of uprooting. The model analysis identified 25 samples as having a high hazard level of uprooting and four samples as having a moderate hazard level of uprooting. These trees, categorized with high and moderate hazard levels of uprooting, should be harvested and pruned, respectively.

Although further analysis, including the incorporation of additional coefficients such as slope and stand spatial density, could enhance the model’s accuracy, the qualitative approach presented here provides a holistic evaluation of tree resistance to uprooting, encompassing both above-ground and below-ground factors. By offering a straightforward and practical assessment tool, this model facilitates decision-making in tree harvest, conservation, and management endeavors.

## Figures and Tables

**Table 1:** Physical properties (mean ± S.E. n = 4) of soil at four depth range.

Depth (cm)	Bulk Density (g·cm^−3^) *	Porosity (%) *	Water Content (%) *	Sand (%) *	Silt (%) *	Clay (%) *
0~15	1.02 ± 0.13	75.35 ± 2.84	42.53 ± 2.41	34.83 ± 2.56	59.23 ± 2.06	5.94 ± 0.81
15~30	0.92 ± 0.15	79.35 ± 3.17	44.17 ± 2.27	31.36 ± 1.73	63.49 ± 3.18	7.66 ± 1.01
30~45	1.17 ± 0.09	63.93 ± 1.55	37.00 ± 2.84	28.41 ± 1.86	60.73 ± 1.47	10.86 ± 1.18
>45	1.24 ± 0.18	70.6 ± 1.89	28.13 ± 3.66	30.02 ± 1.22	50.15 ± 1.36	19.83 ± 1.86

* Mean ± S.E. for the four transects.

**Table 2:** Root plate mass for samples.

Samples	Root Plate Volume (m^3^)	Coarse Root Mass (kg)	Soil Mass in the Root Plate (kg)	Root Plate Mass (kg)	Percentage of Coarse Root Biomass in Root Plate Mass (%)
S1	0.28	40.01	310.29	350.30	11.42
S2	2.00	68.60	2175.40	2243.99	3.06
S3	1.46	64.73	1592.00	1656.73	3.91
S4	2.43	73.43	2652.98	2726.41	2.69
S5	2.44	89.29	2659.25	2748.54	3.25
S6	1.14	59.35	1246.59	1305.94	4.54
S7	2.55	105.22	2775.02	2880.24	3.65
S8	2.55	96.67	2775.02	2871.69	3.37
S9	3.77	111.02	4104.98	4216.00	2.63
S10	3.39	132.07	3695.04	3827.11	3.45
S11	3.81	142.63	4150.04	4292.67	3.32
S12	1.54	57.17	1675.97	1733.14	3.30
S13	2.04	70.78	2221.96	2292.75	3.09
S14	2.15	60.92	2344.52	2405.44	2.53
S15	2.55	78.24	2775.02	2853.26	2.74
S16	0.92	42.40	998.06	1040.45	4.07
S17	0.61	27.98	662.34	690.33	4.05
S18	2.21	68.78	2412.05	2480.83	2.77
S19	0.28	17.57	302.04	319.62	5.50
S20	2.29	70.14	2498.92	2569.06	2.73
Mean ± S.E.	2.02 ± 0.23	73.85 ± 7.16	2201.38 ± 250.09	2275.26 ± 256.79	3.80 ± 0.43
S.D.	1.03	32.02	1118.43	1148.4	1.94
K1	0.46	32.96	502.35	535.31	6.16
K2	1.43	40.01	1553.62	1593.64	2.51
K3	3.21	434.70	3504.05	3938.76	11.04
K4	0.30	23.66	322.81	346.46	6.83
K5	2.08	181.46	2265.70	2447.17	7.42
K6	4.18	1149.48	4556.20	5705.68	20.15
K7	1.95	75.64	2125.94	2201.58	3.44
K8	3.05	219.99	3329.27	3549.27	6.20
K9	3.06	250.79	3338.74	3589.53	6.99
K10	4.00	593.40	4360.00	4953.40	11.98
K11	4.16	1116.47	4534.40	5650.87	19.76
K12	3.45	497.85	3760.50	4258.35	11.69
K13	0.99	36.76	1079.50	1116.26	3.29
K14	0.64	34.18	695.34	729.52	4.69
K15	1.45	40.77	1575.18	1615.94	2.52
K16	0.57	29.37	624.60	653.97	4.49
K17	1.85	47.16	2018.83	2065.98	2.28
K18	0.66	35.86	714.07	749.93	4.78
K19	0.67	40.01	733.72	773.73	5.17
K20	1.76	41.03	1919.08	1960.11	2.09
Mean ± S.E.	2.00 ± 0.30	246.08 ± 77.98	2175.70 ± 324.68	2421.77 ± 393.11	7.17 ± 1.18
S.D.	1.33	348.73	1452.00	1758.04	5.30

**Table 3:** The average morphological characteristics of uprooted spruce and Korean pine.

Species	DBH (cm)	Tree Height (m)	Height to Crown Base (m)	Average Crown Width (m)	Root Plate Volume (m^3^)	Root Plate Mass (kg)
Spruce	28.60 ± 14.50	19.73 ± 8.18	11.15 ± 0.65	6.54 ± 1.66	4.24 ± 1.72	3407.56 ± 137.51
Korean pine	45.18 ± 11.90	24.30 ± 5.14	12.39 ± 0.62	7.71 ± 2.04	10.02 ± 2.04	1624.99 ± 248.14

**Table 4:** Results of $\Delta^{\prime }$ and hazard level of uprooting.

Sample	$\Delta^{\prime }$	Hazard Level of Uprooting
S1	1.18	High
S2	1.01	High
S3	5.32	High
S4	0.17	High
S5	1.51	Low
S6	7.84	Low
S7	0.98	High
S8	2.73	Low
S9	1.40	Moderate
S10	2.82	Low
S11	1.32	Moderate
S12	1.65	Low
S13	0.70	High
S14	0.47	High
S15	1.10	High
S16	0.33	High
S17	2.15	Low
S18	0.31	High
S19	0.70	High
S20	0.43	High
K1	0.83	High
K2	0.90	High
K3	0.71	High
K4	1.72	Low
K5	1.13	High
K6	0.69	High
K7	0.89	High
K8	1.13	High
K9	0.81	High
K10	0.86	High
K11	0.77	High
K12	0.72	High
K13	1.89	Low
K14	1.59	Low
K15	2.39	Low
K16	1.56	Low
K17	0.96	High
K18	1.38	Moderate
K19	0.69	High
K20	1.38	Moderate

**Table 5:** Above ground morphological characteristics of the samples.

Tree Number	DBH (cm)	Height to Crown Base (m)	Tree Height (m)	Crown Width		Average Crown Width (m)
				East-West (m)	North-South (m)	
S1	26.7	3.7	7.8	5.2	5.8	5.5
S2	34.3	6.6	12.9	7.4	6.9	7.2
S3	32.8	5.3	9.6	8.3	4.4	6.4
S4	34.8	6.1	13.4	8.2	8.7	8.5
S5	37.8	7.0	14.1	7.2	5.0	6.1
S6	31.7	5.1	8.4	8.2	9.6	8.9
S7	39.3	6.0	11.8	11.8	12.6	12.2
S8	37.8	5.5	10.2	11.3	8.2	9.8
S9	39.8	5.7	11.8	11.2	12.5	11.9
S10	42.8	6.4	12.0	10.2	8.8	9.5
S11	47.0	11.6	19.3	12.3	8.3	10.3
S12	34.1	10.9	17.8	6.3	7.8	7.1
S13	37.3	12.6	17.4	7.2	4.6	5.9
S14	35.2	11.9	16.9	5.3	6.2	5.8
S15	38.5	12.3	15.9	5.8	9.4	7.6
S16	30.0	8.7	14.9	6.4	8.3	7.4
S17	26.6	10.6	17.1	2.7	3.1	2.9
S18	36.8	12.2	18.6	3.3	10.2	6.8
S19	22.6	10.3	17.6	1.8	4.1	3.0
S20	36.1	9.7	18.8	8.3	11.5	9.9
Mean ± S.E.	35.1 ± 1.28	8.41 ± 0.66	14.32 ± 0.81	7.42 ± 0.67	7.8 ± 0.62	7.64 ± 0.57
S.D.	5.71	2.95	3.63	2.97	2.77	2.53
K1	25.1	7.3	16.7	4.1	3.7	3.9
K2	27.2	12.6	29.1	5.8	6.7	6.3
K3	64.5	16.5	35.6	8.6	8.7	8.7
K4	22.3	7.7	16.6	6.4	6.4	6.4
K5	46.8	11.8	24.2	4.8	8.3	6.6
K6	91.7	18.3	30.9	12.4	14.6	13.5
K7	34.3	14.6	29.7	8.6	8.2	8.4
K8	50.5	16.8	24.8	7.6	9.8	8.7
K9	52.9	16.2	33.4	12.8	13.6	13.2
K10	72.0	14.9	33.8	9.0	9.6	9.3
K11	90.8	18.9	35.9	14.7	15.9	15.3
K12	68.2	23.7	36.2	11.4	10.2	10.8
K13	26.1	7.2	16.2	4.1	5.6	4.9
K14	25.5	8.4	16.8	7.3	5.6	6.5
K15	27.2	8.9	14.9	5.8	6.4	6.1
K16	24.2	9.4	15.6	2.9	4.7	3.8
K17	28.8	11.4	17.3	6.9	5.2	6.1
K18	26.0	9.4	17.2	9.2	7.9	8.6
K19	26.9	7.1	17.2	8.6	7.8	8.2
K20	27.2	7.9	16.5	8.2	9.6	8.9
Mean ± S.E.	42.9 ± 5.14	12.5 ± 1.07	23.9 ± 1.84	8.0 ± 0.69	8.4 ± 0.73	8.2 ± 0.69
S.D.	22.98	4.80	8.22	3.10	3.27	3.11

S: spruce, K: Korean pine.

## Data Availability

Data is contained within the article.
